# The Interim Report of the Committee of Inquiry

**Published:** 1916-07-15

**Authors:** 


### NATIONAL INSURANCE ACT DEVELOPMENTS.

### The Interim Report of the Committee of Inquiry.

In THE HOSPITAL for June 17 (p. 261) we gave the main outlines of the Interim Report of the Committee of Inquiry. We pointed out that the Committee had proved the inadequacy of the present benefit funds to provide for all the benefits for which they are now liable, and we further indicated that the proposals made by the Committee whereby this state of affairs is to be remedied involve a readjustment of the sinking fund contributions. The result of this readjustment will be to postpone for many years—possibly as many as twenty—the period when persons entering insurance at the age of sixteen years will receive the full advantage, in the form of additional or increased benefits, of the contribution of the State. For not until the sinking fund has accumulated to such a sum as will liquidate the initial book reserves, set up for the purpose of enabling the first entrants into insurance to be given the benefits applicable to the age of sixteen years without regard to their actual ages at entry, will the contribution of the State be available for the purpose of augmenting the present benefits, which at the age of sixteen years are provided by the joint contributions of the employer and the insured person.

The Committee endeavoured to show that this postponement of the full advantages of the Act would not in general inflict any hardship upon insured persons, and would certainly not involve any charge of breach of contract on the part of the Government. The argument used was briefly this, that postponement of additional benefits, which could only be granted in any case as directed by Parliament, was more than compensated by the greater stability that would be imparted to the immediate benefit funds by the adoption of their proposals.

### THE DETAILS OF THE SCHEME.

In order, therefore, to arrive at an independent judgment in the matter, it is necessary to examine the details of the proposed application of the moneys deflected from sinking fund purposes. Only by such an investigation is it possible to arrive at a proper conclusion as to the propriety of the "raid on the sinking fund"—as the general idea of their proposals has been described.

The details of the scheme to be fully appreciated demand a considerable amount of technical knowledge and an acquaintance with the practical administration of national insurance finance which few possess, and which, one may say with confidence, will not be possessed even by a large proportion of the members of Parliament who will sooner or later be called upon to decide if the scheme is to be actually adopted. Moreover, the Interim Report, though doubtless admirably written and exceedingly clear to those whose work includes the administration of approved society funds, is a document which cannot be read hurriedly, and in the circumstances it is more than probable that many of its most important points will be unheeded. Nevertheless, the careful reading of the Report will well repay the effort, and in order to assist in popularising the details of the scheme, which must almost immediately be brought forward in Parliament, we endeavour in this article to set forth in a simple manner the more important recommendations of the Committee of Inquiry on points of detail regarding the application of the sinking fund moneys to immediate purposes.

### NEARLY TWO MILLION POUNDS A YEAR.

As at present constituted under the National Insurance Act, 1911, the sinking fund is made up of $1\frac{5}{9}$d. from each contribution paid by male insured persons, being two-ninths of 7d., and $1\frac{1}{2}$d. from each contribution paid by, or on behalf of, each female insured person, being one-quarter of 6d. The net result of the proposals made by the Committee will reduce the amounts above specified to 1d. in the case of men, and $\frac{3}{4}$d. in the case of women per contribution. Thus the amount allotted to the sinking fund is proposed to be reduced by nearly 36 per cent. for men and by 50 per cent. for women. When set out in this way it is easy to see what a substantial difference must necessarily be made by the adoption of the proposals to the term of years that must expire before the sinking fund accomplishes its purpose.

It is not clear at first why the reduction should be greater proportionately for women than for men, but it is explained in the Report that while there is a common sinking fund applicable to both men and women's insurances the reserves for the women's benefits are, on the whole, only about 70 per cent. of those applicable to men, and that under the existing plan the sinking fund contribution of women is too high proportionately to that of men.

The reductions in the amounts—namely, $\frac{5}{9}$d. and $\frac{3}{4}$d.—to one unaccustomed to figures may appear of little moment, but so large is the national insurance scheme that these seemingly small differences in the weekly allocation to sinking fund are estimated to amount to no less than £1,800,000 a year on the basis of the present membership, and as the numbers grow it is likely that in a short while the difference will exceed two million pounds a year.

### THE ALLOCATION OF THE FUND.

The scheme for application of these funds, diverted from future to present objects, is set out under five headings, which provide for: (1) An immediate increase in the income available to societies for the payment of benefits to women generally; (2) further special provision for claims in respect of married women; (3) a special reserve fund to meet, if necessary, the indirect but possibly prolonged effects of war service upon the general sickness rate of the male insured population; (4) additional moneys to meet contingent liabilities which may arise in any society owing to excess of claims for sickness, disablement, or maternity benefits, such moneys to be immediately available in cases of deficiency on valuation; and (5) further funds to meet special risks incidental to an abnormally constituted society, arising, *e.g.*, from inclusion of an excessive proportion of those engaged in hazardous occupations.

We need not show in detail the respective amounts, or proportions of the total of £1,800,000, which it is proposed to allocate to the above-mentioned funds. It is, however, of interest to note the general principles underlying the apportionment.

### Addition to Women's Benefit Funds.

The first feature to attract attention is the recommendation that a direct addition should be made to the current income of the benefit funds in respect of all women's insurances. This is a direct and very practical recognition of the inadequacy of the amount at present at disposal. But over and beyond this direct addition there is to be a "Women's Equalisation Fund," the primary object of which is to provide a means for relieving those approved societies which, through having an undue proportion of married members, experience higher claims for sickness and maternity benefits than those whose membership is normal in regard to the marital condition. This proposed fund is to be of a central character, applicable to the whole of the United Kingdom, and will, presumably, be under the control of the Joint Committee of the Insurance Commissions. The ultimate basis for the disposal of the money is twofold, one part being divided among societies in proportion to the number of married women included in their membership, and the other part as a grant in respect of maternity benefit claims.

### Effects of the War.

Among the unexpected contingencies at the time the original estimates were made we now have to include those arising out of the present war. How far the war will ultimately affect national insurance finance is even now little more than a speculation. It is clear that in some respects there may actually be savings to approved societies, by the release of reserves for example, but on the other hand it is probable that the hardships which so many of our sailors and soldiers have had to undergo will have a material effect on their general health and will cause a greater strain on the funds of the societies when they once more return to their civil occupations than was originally anticipated. In these circumstances it is certainly prudent, and sound finance, to set aside a portion of the released moneys to provide a fund to be known as the "Men's Special Reserve Fund," out of which the excess claims due indirectly to the war may be met.

We think it sufficiently important to draw attention in this connection to the actual words employed by the Committee of Inquiry, when pointing out that the object of the "Men's Special Reserve Fund" is to meet only claims indirectly due to the war. They say ". . . we could not be a party to any proposal which involved placing upon those [namely, national insurance] funds a burden which should properly be borne by the community at large."

### The Contingencies and Special Risks Funds.

The two remaining items on the list are to be known as the "Contingencies Fund" and the "Special Risks Fund." The "Contingencies Fund" is to be considered as a means of equalising casual variations in the claims of a society. The fund is to be divided among the societies in much the same manner as the "Men's Special Reserve Fund" in proportion to membership and other like considerations under due regulations as to its disposal. The "Special Risks Fund" on the other hand, being designed to correct inequalities in the composition of membership of societies, will be a central fund applicable to the whole of the United Kingdom from which grants will be made as and when required. Thus claims for assistance from this fund will have to be investigated before relief is given.

### An Emergency Measure Only.

The Report shows that the Committee had to face a difficult problem, but it also shows that they faced it with commendable courage and the recommendations appear to be the best that could be made within the limitations of the terms of reference by which the Committee were bound. It is, doubtless, impossible at the present time to obtain additional grants from the Government for national insurance purposes, but this is really the only way in which the finance can ultimately be placed upon a sound basis, and we cannot look upon the proposals of the Committee of Inquiry, admirable though they are, except as emergency recommendations to enable approved societies to make their first valuations with some prospect of showing a general condition of solvency.

